# Lung transplantation in recipients aged ≥70 years: a single-center experience

**DOI:** 10.1016/j.jhlto.2026.100542

**Published:** 2026-03-20

**Authors:** Jan Jelinek, Tomas Kusnirak, Monika Svorcova, Jaromir Vajter, Jan Balko, Gabriela Holubova, Zuzana Ozaniak Strizova, Pavel Pafko, Rene Novysedlak, Jiri Vachtenheim, Robert Lischke

**Affiliations:** a Prague Lung Transplant Program, 3rd Department of Surgery, First Faculty of Medicine, Charles University and Motol University Hospital, Prague, Czech Republic; b First Faculty of Medicine, Charles University, Prague, Czechia; c Department of Anesthesiology, Resuscitation and Intensive Care Medicine, Second Faculty of Medicine, Charles University and Motol University Hospital, Prague, Czech Republic; d Department of Pathology and Molecular Medicine, Second Faculty of Medicine, Charles University and Motol University Hospital, Prague, Czech Republic; e Department of Immunology, Second Faculty of Medicine, Charles University and Motol University Hospital, Prague, Czech Republic

**Keywords:** Lung transplantation, Organ donation, Septuagenarian lung recipients, Primary graft dysfunction, Transplant survival

## Abstract

The suitability of lung transplantation (LTx) in recipients aged ≥70 years remains debated, despite reports of acceptable outcomes in selected elderly patients among other solid-organ transplantations. We retrospectively analyzed all LTx procedures performed in this age group within the Prague Lung Transplant Program between January 2012 and July 2025. Twelve patients aged ≥70 years underwent double (n = 5) or single (n = 7) LTx. The median age was 70.3 years. Indications were interstitial pulmonary disease (n = 7) and chronic obstructive pulmonary disease (n = 5). Median waiting time was 199.5 days. Primary graft dysfunction grade 2 occurred in 2 patients, with no cases of grade 3. Median ICU and hospital lengths of stay were 10 and 21.5 days, respectively. One-month and 1-year survival rates were 91.7% and 73%. These data indicate that LTx in carefully selected septuagenarian recipients can achieve favorable short-term outcomes.

Lung transplantation (LTx) represents a definitive therapeutic option for selected patients with end-stage pulmonary disease. As life expectancy increases, an expanding proportion of transplant candidates are aged ≥70 years; however, advanced age remains a relative contraindication due to comorbidities and concerns regarding outcome. The aim of this study was to evaluate perioperative courses and outcomes of LTx in recipients aged ≥70 years.

## Methods

A retrospective study was conducted at Motol University Hospital (Prague, Czech Republic). All patients aged ≥70 years who underwent LTx between January 2012 and July 2025 were included. Data were collected from institutional databases, with follow-up censored on October 31, 2025. Approval from local institutional review board was granted. The study is compliant with the International Society for Heart and Lung Transplantation (ISHLT) Ethics statement.

Donor and preservation characteristics were: age, sex, body mass index (BMI), cause of death, preservation method, total ischemic time, and last donor partial oxygen pressure divided by the fraction of inspired oxygen in arterial blood gases (PaO_2_/FiO_2_).

Recipient characteristics included in the study are age, sex, BMI, indication for transplantation, mean pulmonary arterial pressure (mPAP), time on waiting list, transplant type, intraoperative blood loss, duration of surgery, surgical approach, and use of extracorporeal membrane oxygenation (ECMO). Outcomes assessed were length of intensive care unit (ICU) and hospital stay, primary graft dysfunction (PGD), acute cellular rejection (ACR), pulmonary function, and survival.

PGD was assessed using daily chest radiography and arterial blood gas analysis and graded according to the 2016 ISHLT consensus statement.^1^ ACR diagnosis relied on transbronchial biopsy samples evaluated by an experienced pathologist.^2^

Continuous variables were reported as median (minimum–maximum) and categorical variables as number (percentage).

Further details regarding surgical technique and intraoperative management are provided in the Supplementary Methods.

## Results

During the study period, 598 LTx were performed, of which 12 patients were aged ≥70 years. Seven (58%) underwent single-LTx. Donor, recipient, intraoperative characteristics, and outcomes are summarized in Table 1, Table 2. Recipient, intraoperative characteristics, and outcomes stratified by transplant type (single vs bilateral lung transplantation) are provided in Supplementary Table S1**.**

**Table 1:** Donor and Lung Preservation Characteristics

Donor and lung preservation characteristics		Number of missing values
Age (years)	53.5 (26-71)	-
*Sex*		
Male, n (%)	5 (41.7)	-
Female, n (%)	7 (58.3)	-
BMI	24.2 (18.8-35.2)	1
*Cause of death*		1
Intracerebral bleeding, n (%)	5 (41.7)	-
Trauma, n (%)	2 (16.7)	-
Ischemic stroke, n (%)	1 (8.3)	-
Asphyxia, n (%)	1 (8.3)	-
Cardiac arrest, n (%)	1 (8.3)	-
Intoxication, n (%)	1 (8.3)	-
*Storage method*		
Ice box, n (%)	10 (83.3)	-
Cold storage 10°C, n (%)	1 (8.3)	-
PARAGONIX LUNGguard, n (%)	1 (8.3)	-
*Total ischemic time (minutes)*		
First lung	232 (163-799)	-
Second lung	310 (265-407)	7
Last donor PaO2/FiO2 (mmHg)	384 (331-465)	2

BMI, body mass index; PaO₂/FiO₂, ratio of arterial oxygen partial pressure to fraction of inspired oxygen. Continuous variables are reported as median (minimum–maximum) and categorical variables as number (percentage)

**Table 2:** Recipient and Intraoperative Characteristics and Outcomes

Recipient and intraoperative characteristics		Number of missing values
Age (years)	70.3 (70.0-71.6)	-
*Sex*		
Male, n (%)	6 (50)	-
Female, n (%)	6 (50)	-
BMI (kg/m^2^)	26.6 (18.9-29.4)	-
*Indication*		
ILD, n (%)	7 (58.3)	-
COPD, n (%)	5 (41.7)	-
mPAP (mmHg)	26 (17-43)	1
Time on WL (days)	199.5 (16-726)	-
*Type*		
SLTx, n (%)	7 (58.3)	-
right, n (%)	3 (43)	-
left, n (%)	4 (57)	-
DLTx, n (%)	5 (41.7)	-
Blood loss (ml)	650 (200-1500)	-
Duration of transplant (min)	273 (153-420)	-
*Surgical access*		
Anterolateral thoracotomy, n (%)	7 (58.3)	-
Clamshell thoracotomy, n (%)	5 (41.7)	-
Intraoperative ECMO, n (%)	5 (41.7)	-
*Outcomes*		
ICU length of stay (days)	10 (5-32)	-
Hospital length of stay (days)	21.5 (15-42)	-
PGD 2 within 72 h, n (%)	3 (25)	1
PGD 2 at 72 h, n (%)	2 (16.7)	1
PGD 3 within 72 h, n (%)	0 (0)	1
ACR at 1 month, n	3	N/A
ACR at 6 months, n	2	N/A
ACR at 1 year, n	2	N/A
FEV1 at 1 month (%)	84 (53-100)	3
FEV1 at 6 months (%)	73.9 (55-132)	4
FEV1 at 1 year (%)	80.3 (46-103)	6
Alive at 1 month, n (%)	12 (100)	-
Alive at 6 months, n (%)	9 (75)	-
Alive at 1 year, n (%)	7 (58.3)	2
Survival (days, follow-up date October 31st, 2025)	683 (74-1171)	-

ACR, acute cellular rejection; BMI, body mass index; COPD, chronic obstructive pulmonary disease; DLCO, diffusing capacity of the lungs for carbon monoxide; DLTx, double (bilateral) lung transplantation; ECMO, extracorporeal membrane oxygenation; FEV₁, forced expiratory volume in 1 sec; FVC, forced vital capacity; ICU, intensive care unit; ILD, interstitial lung disease; LTx, lung transplantation; mPAP, mean pulmonary arterial pressure; PGD, primary graft dysfunction; SLTx, single-lung transplantation; WL, waiting list. Continuous variables are reported as median (minimum–maximum) and categorical variables as number (percentage). In this age group, surveillance biopsies for acute cellular rejection (ACR) are not routinely performed in the absence of clinical suspicion; therefore, missing data and percentages for ACR are not reported

The median donor age was 53.5 years (26-71) and 7 (58%) donors were female. Among (all brain death) donors, intracerebral hemorrhage was the most frequent cause of death (42%). Lungs were predominantly preserved in an ice box (10, 83.3%). Median total ischemic time of the first implanted lung was 232 min (163-799), in bilateral procedures, the second-lung total ischemic time was 310 min (265-407).

The median recipient age was 70.3 years (70-71.6), and 6 (50%) of the recipients were female. Indications were chronic obstructive pulmonary disease (COPD) (n = 5) and interstitial lung disease (ILD) (n = 7). Median pre-LTx mPAP was 26 mmHg (16-43). Group 3 pulmonary hypertension was present in 8(66%) patients, while 3(25%) had normal pressures.^3^ Median waiting-list time was 199.5 days (16-726). Intraoperative ECMO was used in 5(42%) procedures. Median operating time was 273 min (153-420). When stratified by transplant type, operative time was 200 min (153-275) for single-LTx and 375 min (338-420) for bilateral LTx. Median blood loss was 650 ml (200-1500).

All bilateral LTx recipients required intraoperative ECMO (median 255 min, 205-260), whereas none of the single-LTx recipients did. Median intensive care unit (ICU) stay, and total hospital stay were 10(5-32) and 21.5 days (11-42), respectively. Three (25%) patients developed PGD 2 within 72 hours, and none developed PGD 3.

**Figure 1:**
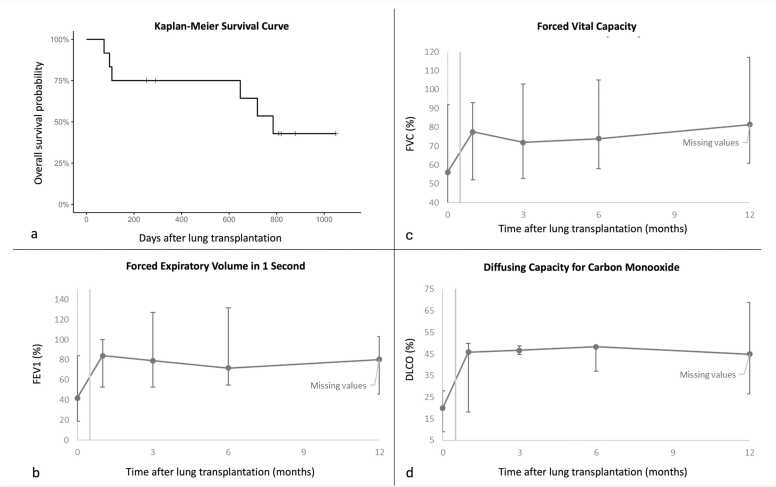
Post-transplant survival and pulmonary function over 12 months. (**a**) Kaplan–Meier survival curve illustrating overall survival of the entire cohort after lung transplantation. (**b**) Forced expiratory volume in 1 s (FEV₁), (**c**) Forced vital capacity (FVC), and (**d**) Diffusing capacity for carbon monoxide (DLCO) expressed as % predicted. Values represent medians with ranges. Pulmonary function improved rapidly after transplantation and remained stable to 12 months.

Lung function improved by 12 months. Median forced expiratory volume in 1 sec (FEV1) increased from pre-transplant 42% to 80.3% predicted, forced vital capacity (FVC) from 56.2% to 81.5% predicted, and diffusing capacity of the lungs for carbon monoxide (DLCO) from 20% to 45% predicted (Figure 1 **b-c**).

All patients survived 1 month. One (8.3%) patient died within 3 months from pneumonia. Two (17%) additional deaths occurred within 6 months, due to pulmonary embolism on day 97 and stroke on day 107. At the end of the follow-up (October 31, 2025), median overall survival was 683 days (74-1171), with 1-year survival 73%. One patient had not yet reached 1 year. The Kaplan–Meier survival curve for the entire cohort is shown in Figure 1a, with curves comparing single and double lung transplantation provided in Supplementary Fig. S1. Median 1-year pulmonary function showed an FVC of 81.5% predicted and FEV1 of 80% predicted.

## Discussion

With rising life expectancy, surgical procedures in elderly require careful evaluation. In thoracic surgery, the traditional concern of increased perioperative risk in older adults has been challenged by recent data. Kirk et al. demonstrated that patients aged ≥80 years had morbidity comparable to younger controls (30% vs 20.8%) and no significant increase in 30-day mortality (1.67% vs 0.17%), indicating that advanced age alone does not predict poorer outcomes when selection is rigorous.^4^

Similar findings are reported across solid-organ transplantation. In heart transplantation, Henricksen et al*.* found only a minor reduction in 1-year survival among recipients ≥70 years (87.5%) compared with those <60 years (91.1%) and 60-69 years (88.4%), while 5-year survival remained high.^5^ Boesmueller et al*.* likewise demonstrated favorable outcomes in kidney recipients >70 years, with 1-year survival of 95% and death-censored graft survival of 100%, similar to younger controls.^6^

Zhou et al. reported that recipients aged ≥70 years were more likely to undergo LTx and less likely to die on the waitlist than younger candidates.^7^ Perioperative outcomes were favorable, with lower rates of acute rejection (6.7% vs 7.4% in patients aged 60%-69% and 9.2% in those aged 18-59 years) and prolonged intubation (21.7% vs 27.4% and 34.5%, respectively). One-year survival reached 83.6%, compared with 89.3% in recipients aged 18%-59% and 86.5% in those aged 60-69 years.^7^

In our cohort, all bilateral lung transplantations were performed with intraoperative ECMO support, whereas none of the single-lung procedures utilized ECMO. Avoidance of extracorporeal circulation in single-LTx may theoretically attenuate systemic inflammatory response, vasodilation, and platelet dysfunction, potentially reducing complications such as bleeding or vasoplegia.^8^, ^9^ Nevertheless, this observation should be interpreted cautiously, as ECMO utilization and the choice of transplant procedure are largely influenced by procedural complexity, patient selection, and institutional practice.

The 3-month and 1-year survival rates (91.7% and 73%) are comparable to those reported in unselected adult cohorts. When contrasted with the report by Thabut et Mal (2017), which described 3-month and 1-year survival of 89% and 80%, respectively, our early outcomes appear favorable.^10^ According to the ISHLT Registry, 1-year survival in adult LTx recipients has steadily improved, increasing from 75.8%(1992-2000) to 81.7%(2001-2009), 86.8%(2010-2017), and 88.4%(2018-2023).^11^ However, the median overall survival of 683 days in our cohort warrants particular attention and underscores the need for further optimization of mid- to long-term outcomes. These findings should also be interpreted in the context of recipient age and comorbidity burden, which may contribute to lower early survival even in carefully selected elderly patients.

Our findings should be interpreted in light of several limitations. This study represents a retrospective single-center experience involving a small number of highly selected patients, which limits statistical inference and generalizability. In addition, the cohort consisted exclusively of COPD and ILD patients, and none had primary pulmonary hypertension.

## Conclusion

Lung transplantation in septuagenarian recipients should be reserved for carefully selected patients and performed in experienced centers.

## CRediT Authorship Contribution Statement

Conceptualization: JJ, TK, ZOS, RN, JVJr. Data curation: JJ, TK, MS, JV, JB, RN. Formal analysis: JJ, TK, RN. Methodology: GH, RN, JVJr. Supervision: ZOS, PP, RL, RN, JVJr. Visualization: JJ, TK, RN. Writing - original draft: JJ, TK, RN. Writing - review & editing: JJ, TK, MS, JV, JB, GH, ZOS, PP, RL, RN, JVJr. All authors read and approved the final manuscript.

## Declaration of Generative AI and AI-assisted technologies in the writing process

During the preparation of this work, the authors used ChatGPT (OpenAI) in order to improve readability and language. After using this tool, the authors reviewed and edited the content as needed and take full responsibility for the content of the publication.

## Data availability statement

Data can be obtained from the corresponding author upon request.

## Declaration of Competing Interest

The authors declare that they have no known competing financial interests or personal relationships that could have appeared to influence the work reported in this paper.

## Footnotes

Appendix A Supplementary data associated with this article can be found in the online version at doi:10.1016/j.jhlto.2026.100542.
