# Supplementary material for: Lung transplantation in recipients aged ≥70 years: a single-center experience
Source: JHLT Open. 2026 Mar 20;12:100542. doi: 10.1016/j.jhlto.2026.100542 (PMC13091370; doi:10.1016/j.jhlto.2026.100542)
Supplement: Supplementary file 1 — Supplementary material [file mmc1.docx]

**Lung Transplantation in Recipients Aged ≥70 Years:**

**A Single-Center Experience**

*Supplementary Figure*

Jan Jelinek, MD^a*^, Tomas Kusnirak^b*^, Monika Svorcova, MD^a^, Jaromir Vajter, MD, PhD^c^, Jan Balko, MD, PhD^d^, Gabriela Holubova, MD^c^, Zuzana Ozaniak Strizova, MD, PhD^e^, Pavel Pafko, MD, PhD^a^, Rene Novysedlak, MD, PhD^a+^, Jiri Vachtenheim Jr, MD, PhD^a^, Robert Lischke, MD, PhD^a^

^a^ Prague Lung Transplant Program, 3rd Department of Surgery, First Faculty of Medicine, Charles University and Motol University Hospital, Prague, Czech Republic

^b^ First Faculty of Medicine, Charles University

^c^ Department of Anesthesiology, Resuscitation and Intensive Care Medicine, Second Faculty of Medicine, Charles University and Motol University Hospital, Prague, Czech Republic

^d^ Department of Pathology and Molecular Medicine, Second Faculty of Medicine, Charles University and Motol University Hospital, Prague, Czech Republic

^e^ Department of Immunology, Second Faculty of Medicine, Charles University and Motol University Hospital, Prague, Czech Republic

* Authors contributed equally.

^+^**Corresponding Author**

Dr. René Novysedlák (MD, PhD)

Prague Lung Transplant Program

3^rd^ Department of Surgery, Motol University Hospital

First Faculty of Medicine, Charles University

V Uvalu 84

15006 Prague

Tel.: +420 608 931 829

LinkedIn: [www.linkedin.com/](http://www.linkedin.com/in/laurens-ceulemans-1190a7a1)in/rnovysedlak

Twitter: @ReneNovysedlak

E-mail: [rene.novysedlak@lf1.cuni.cz](mailto:rene.novysedlak@lf1.cuni.cz)


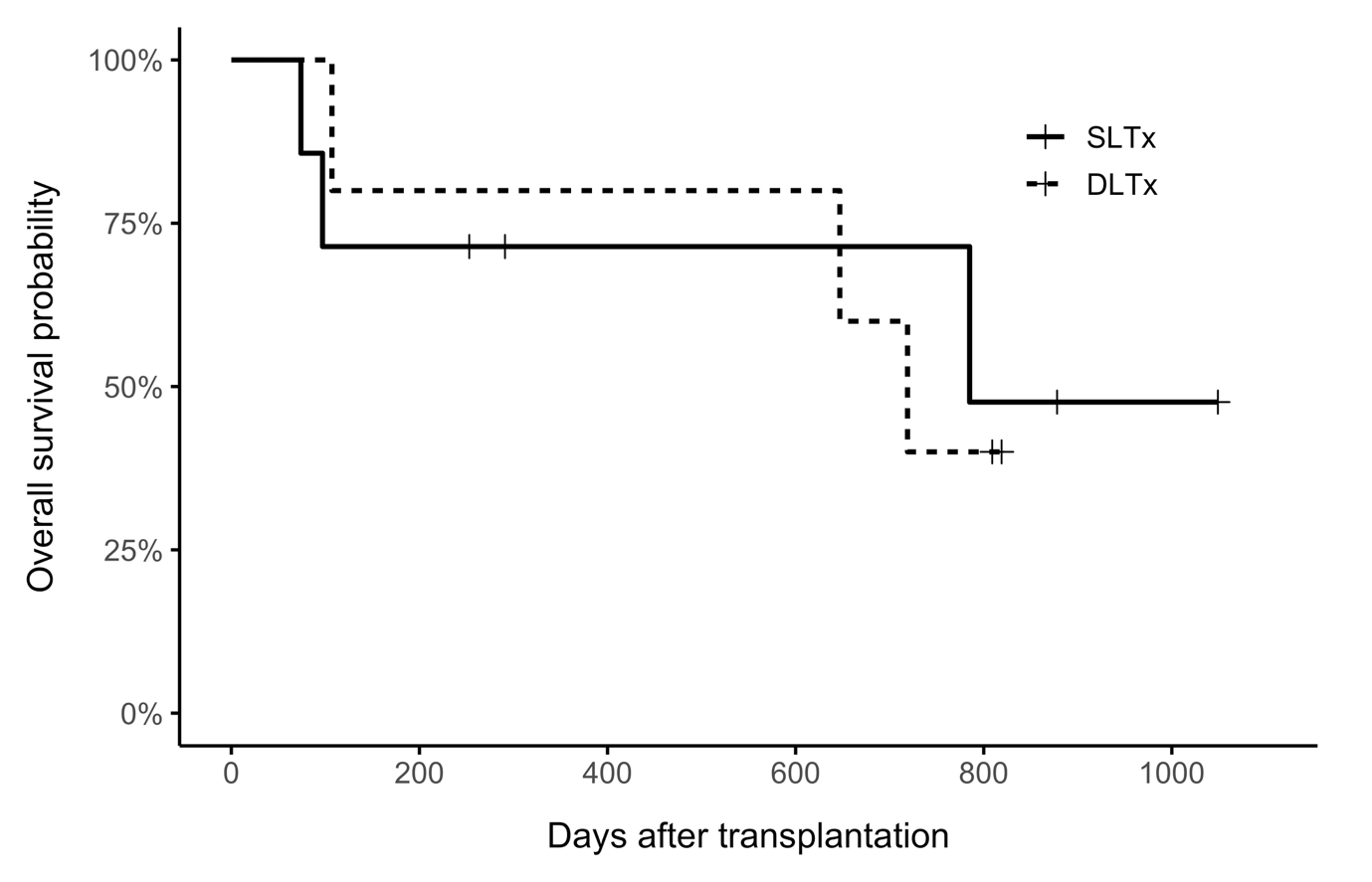


**Supplementary Figure S1.** Kaplan–Meier survival curves according to transplant type. Kaplan–Meier estimates of overall survival after lung transplantation in recipients aged ≥70 years stratified by transplant type. Solid line represents single-lung transplantation (SLTx) and dashed line represents bilateral lung transplantation (DLTx). Tick marks indicate censored observations. Due to the small subgroup sizes (SLTx n=7; DLTx n=5), this comparison is presented for descriptive purposes only and should be interpreted with caution.
