# Supplementary material for: Lung transplantation in recipients aged ≥70 years: a single-center experience
Source: JHLT Open. 2026 Mar 20;12:100542. doi: 10.1016/j.jhlto.2026.100542 (PMC13091370; doi:10.1016/j.jhlto.2026.100542)
Supplement: Supplementary file 2 — Supplementary material [file mmc2.docx]

**Lung Transplantation in Recipients Aged ≥70 Years:**

**A Single-Center Experience**

*Supplementary Methods*

Jan Jelinek, MD^a*^, Tomas Kusnirak^b*^, Monika Svorcova, MD^a^, Jaromir Vajter, MD, PhD^c^, Jan Balko, MD, PhD^d^, Gabriela Holubova, MD^c^, Zuzana Ozaniak Strizova, MD, PhD^e^, Pavel Pafko, MD, PhD^a^, Rene Novysedlak, MD, PhD^a+^, Jiri Vachtenheim Jr, MD, PhD^a^, Robert Lischke, MD, PhD^a^

^a^ Prague Lung Transplant Program, 3rd Department of Surgery, First Faculty of Medicine, Charles University and Motol University Hospital, Prague, Czech Republic

^b^ First Faculty of Medicine, Charles University

^c^ Department of Anesthesiology, Resuscitation and Intensive Care Medicine, Second Faculty of Medicine, Charles University and Motol University Hospital, Prague, Czech Republic

^d^ Department of Pathology and Molecular Medicine, Second Faculty of Medicine, Charles University and Motol University Hospital, Prague, Czech Republic

^e^ Department of Immunology, Second Faculty of Medicine, Charles University and Motol University Hospital, Prague, Czech Republic

* Authors contributed equally.

^+^**Corresponding Author**

Dr. René Novysedlák (MD, PhD)

Prague Lung Transplant Program

3^rd^ Department of Surgery, Motol University Hospital

First Faculty of Medicine, Charles University

V Uvalu 84

15006 Prague

Tel.: +420 608 931 829

LinkedIn: [www.linkedin.com/](http://www.linkedin.com/in/laurens-ceulemans-1190a7a1)in/rnovysedlak

Twitter: @ReneNovysedlak

E-mail: [rene.novysedlak@lf1.cuni.cz](mailto:rene.novysedlak@lf1.cuni.cz)

**Surgical Technique and Intraoperative Management**

Extracorporeal life support (ECLS) was not routinely planned for single lung transplantation; however, peripheral veno-arterial extracorporeal membrane oxygenation (ECMO) was available in all cases and could be rapidly instituted if clinically indicated. The decision to proceed without planned ECLS was based on preoperative assessment and intraoperative monitoring. In all cases, both surgical and perfusion teams were prepared for immediate ECMO initiation if required.

Size mismatch between donor and recipient lungs was managed by tailoring of the donor lung, most commonly by right-sided volume reduction, typically through resection of the middle lobe. Diaphragmatic plication was not routinely performed and was reserved for cases of clinically significant phrenic nerve dysfunction, which were not present in this cohort.

Bilateral lung transplantation was performed using a sequential approach. Both recipient hila were initially dissected and prepared for explantation. Following implantation and reperfusion of the first lung, attention was turned to explantation of the contralateral native lung, followed by completion of hilar preparation and implantation of the second graft. These steps were performed in a streamlined and partially parallelized manner. Procedures were typically performed by two experienced surgeons, with a dedicated team member preparing the donor lung on the back table in parallel.

Bronchial anastomoses were routinely performed using a single running double-armed 4-0 polydioxanone (PDS) suture. In cases of size mismatch between donor and recipient bronchi, the technique was adapted at the surgeon’s discretion, typically by combining a running suture for the membranous portion with interrupted sutures for the cartilaginous portion, or by using fully interrupted sutures.
