# Supplementary material for: Lung transplantation in recipients aged ≥70 years: a single-center experience
Source: JHLT Open. 2026 Mar 20;12:100542. doi: 10.1016/j.jhlto.2026.100542 (PMC13091370; doi:10.1016/j.jhlto.2026.100542)
Supplement: Supplementary file 3 — Supplementary material [file mmc3.docx]

**Lung Transplantation in Recipients Aged ≥70 Years:**

**A Single-Center Experience**

*brief communication*

**Supplementary Tables**

Jan Jelinek, MD^a*^, Tomas Kusnirak^b*^, Monika Svorcova, MD^a^, Jaromir Vajter, MD, PhD^c^, Jan Balko, MD, PhD^d^, Gabriela Holubova, MD^c^, Zuzana Ozaniak Strizova, MD, PhD^e^, Pavel Pafko, MD, PhD^a^, Rene Novysedlak, MD, PhD^a+^, Jiri Vachtenheim Jr, MD, PhD^a^, Robert Lischke, MD, PhD^a^

^a^ Prague Lung Transplant Program, 3rd Department of Surgery, First Faculty of Medicine, Charles University and Motol University Hospital, Prague, Czech Republic

^b^ First Faculty of Medicine, Charles University and Motol University Hospital, Prague, Czech Republic

^c^ Department of Anesthesiology, Resuscitation and Intensive Care Medicine, Second Faculty of Medicine, Charles University and Motol University Hospital, Prague, Czech Republic

^d^ Department of Pathology and Molecular Medicine, Second Faculty of Medicine, Charles University and Motol University Hospital, Prague, Czech Republic

^e^ Department of Immunology, Second Faculty of Medicine, Charles University and Motol University Hospital, Prague, Czech Republic

* Authors contributed equally.

^+^**Corresponding Author**

Dr. René Novysedlák (MD, PhD)

Prague Lung Transplant Program

3^rd^ Department of Surgery, Motol University Hospital

First Faculty of Medicine, Charles University

V Uvalu 84

15006 Prague

Tel.: +420 608 931 829

LinkedIn: [www.linkedin.com/](http://www.linkedin.com/in/laurens-ceulemans-1190a7a1)in/rnovysedlak

Twitter: @ReneNovysedlak

E-mail: [rene.novysedlak@lf1.cuni.cz](mailto:rene.novysedlak@lf1.cuni.cz)

| ***Recipient and intraoperative characteristics*** | | | Number of missing values |
| --- | --- | --- | --- |
|  | SLTx (n = 7) | DLTx (n = 5) |  |
| Age (years) | 70.29 (70.05 – 71.61) | 70.39 (70.04 – 70.89) | - |
| *Sex* |  |  |  |
| Male, n (%) | 4 (57) | 2 (40) | - |
| Female, n (%) | 3 (43) | 3 (60) | - |
| BMI (kg/m^2^) | 26.85 (18.9 – 28.4) | 26.35 (18.9 – 29.4) | - |
| *Indication* |  |  |  |
| ILD, n (%) | 7 (100) | 0 | - |
| COPD, n (%) | 0 | 5 (100) | - |
| mPAP (mmHg) | 21 (16 – 28) | 27 (21 – 43) | 0 / 1 |
| Time on WL (days) | 79 (17 – 466) | 366 (28 – 726) | - |
| *Type* |  |  |  |
| SLTx, n (%) | 7 (100) | 0 | - |
| right, n (%) | 3 (43) | - | - |
| left, n (%) | 4 (57) | - | - |
| DLTx, n (%) | 0 | 5 (100) | - |
| Blood loss (ml) | 300 (200 – 800) | 1000 (800-1500) | - |
| Duration of transplant (min) | 200 (153 – 275) | 375 (338 – 420) | - |
| *Surgical access* |  |  |  |
| Anterolateral thoracotomy, n (%) | 7 (100) | 0 | - |
| Clamshell thoracotomy, n (%) | 0 | 5 (100) | - |
| Intraoperative ECMO, n (%) | 0 | 5 (100) | - |
| ***Outcomes*** | | |  |
| ICU length of stay (days) | 10 (5 – 12) | 10 (7 – 32) | - |
| Hospital length of stay (days) | 20 (15 – 33) | 27 (17 – 42) | - |
| PGD 2 within 72h, n (%) | 1 (16.7) | 2 (40) | 1 / 0 |
| PGD 2 at 72h, n (%) | 1 (16.7) | 1 (20) | 1 / 0 |
| PGD 3 within 72h, n (%) | 0 | 0 | 1 / 0 |
| ACR at 1 month, n (%) | 0 | 3 | N/A |
| ACR at 6 months, n (%) | 0 | 2 | N/A |
| ACR at 1 year, n (%) | 1 | 1 | N/A |
| FEV1 at 1 month (%) | 68 (52 – 99) | 99 (84 – 100) | 2 / 1 |
| FEV1 at 6 months (%) | 73.9 (71 – 97.9) | 83.5 (55 – 131.5) | 3 / 1 |
| FEV1 at 1 year (%) | 87.8 (72.6 – 102.9) | 77.5 (46 – 101) | 5 / 1 |
| Alive at 1 month, n (%) | 7 (100) | 5 (100) | - |
| Alive at 6 months, n (%) | 5 (71.4) | 4 (80) | - |
| Alive at 1 year, n (%) | 3 (60) | 4 (80) | 2 / 0 |
| Survival (days,  follow-up date October 31^st^, 2025) | 321 (74 – 1079) | 719 (107 – 849) | - |

**Table S1.** Recipient and intraoperative characteristics and outcomes stratified by transplant type (single vs. bilateral lung transplantation). Continuous variables are reported as median (minimum–maximum) and categorical variables as number (percentage). In this age group, surveillance biopsies for acute cellular rejection (ACR) are not routinely performed in the absence of clinical suspicion; therefore, missing data and percentages for ACR are not reported.

Abbreviations: ACR – acute cellular rejection; BMI – body mass index; COPD – chronic obstructive pulmonary disease; DLCO – diffusing capacity of the lungs for carbon monoxide; DLTx – double (bilateral) lung transplantation; ECMO – extracorporeal membrane oxygenation; FEV₁ – forced expiratory volume in one second; FVC – forced vital capacity; ICU – intensive care unit; ILD – interstitial lung disease; LTx – lung transplantation; mPAP – mean pulmonary arterial pressure; PGD – primary graft dysfunction; SLTx – single-lung transplantation; WL – waiting list
